# Short-Term Sulfurous Balneotherapy and Self-Reported Sleep Quality: An Exploratory Retrospective Real-World Pre–Post Observational Study at Terme di Saturnia (Italy)

**DOI:** 10.3390/healthcare14060782

**Published:** 2026-03-19

**Authors:** Elisabetta Ferrara, Manela Scaramuzzino, Giuseppe Balice, Giovanna Murmura, Bruna Sinjari

**Affiliations:** 1 Department of Human Sciences, Law and Economics, Telematic University “Leonardo Da Vinci” (UNIDAV), 66100 Torrevecchia Teatina, Italy; 2 Medical Thermal Center of Saturnia, 58014 Grosseto, Italy; manela.scaramuzzino@gmail.com; 3 Unit of Prosthodontics, Department of Innovative Technologies in Medicine and Dentistry, University “G. d’Annunzio” of Chieti-Pescara, 66100 Chieti, Italy; giuseppe.balice@unich.phd.it (G.B.); giovanna.murmura@unich.it (G.M.); bruna.sinjari@unich.it (B.S.)

**Keywords:** balneotherapy, sulfurous thermal water, insomnia, sleep satisfaction, sleep quality, Oviedo Sleep Questionnaire, real-world evidence, observational study, non-pharmacological intervention, healthcare management

## Abstract

**Background:** Sleep disturbances are highly prevalent, affecting approximately 21% of the European population, with chronic insomnia disorder estimated at 6%. Non-pharmacological alternatives to pharmacotherapy are needed. Sulfurous balneotherapy represents a potential intervention, yet real-world evidence remains limited. **Objective:** To explore changes in self-reported sleep quality following sulfurous balneotherapy at Terme di Saturnia (Italy). **Methods:** Retrospective single-arm observational study of 76 participants (mean age 47.3 years, 54% female) undergoing a 7–12-day consecutive balneotherapy cycle with daily sulfurous thermal water immersion sessions (60–90 min/session). The Oviedo Sleep Questionnaire (OSQ) was administered pre- and post-treatment. Participants were stratified by baseline insomnia severity into Group A (OSQ ≥ 22, n = 47) and Group B (OSQ < 22, n = 29). The primary outcome was change in OSQ insomnia score in Group A. Statistical analysis was performed using the Wilcoxon signed-rank test. **Results:** In Group A, insomnia severity decreased significantly from 26.4 ± 8.3 at baseline to 20.1 ± 7.5 post-treatment (Δ = −6.3, 95% CI: −7.9 to −4.7, *p* < 0.001, r = 0.54). Sleep satisfaction also improved significantly from 3.2 ± 1.1 to 4.6 ± 1.2 (Δ = +1.4, 95% CI: 1.1–1.7, *p* < 0.001, r = 0.60). In Group B, no statistically significant changes were observed, consistent with ceiling effects. However, in an open-ended question, 72.4% (21/29; 95% CI: 54.3–85.3) of Group B participants reported enhanced relaxation during the spa stay. Due to the single-arm observational design without control groups, the observed improvements cannot be distinguished from non-specific factors, including the vacation effect, reduced work-related stress, placebo and expectancy responses, regression to the mean, or the effects of warm water immersion itself independent of sulfurous mineral content. **Conclusions:** This exploratory study documents pre–post improvements in self-reported sleep quality in a cohort undergoing sulfurous balneotherapy during a spa vacation. The absence of control groups and unmeasured confounders precludes causal inferences. Future randomized trials with heated non-mineral water controls are needed to isolate specific therapeutic contributions of sulfurous thermal waters.

## 1. Introduction

Sleep is a complex physiological process fulfilling multiple essential functions, including tissue repair and cellular regeneration, metabolic regulation, and neural plasticity critical for cognitive performance and brain development [1]. Sleep disturbances are highly prevalent, affecting approximately 21% of the European population, with chronic insomnia disorder estimated at 6% [2]. The clinical significance of sleep disturbances extends beyond subjective discomfort and includes associations with depression, hypertension, obesity, diabetes, and increased mortality risk [1,3,4,5]. Occupational studies further illustrate the breadth of these consequences: in a representative sample of shift-working nurses, insomnia was significantly associated with cardiovascular, gastrointestinal, and neuropsychiatric symptoms, underscoring the systemic impact of chronic sleep disruption [6]. Pharmacological interventions remain the most commonly prescribed treatment for sleep disorders. However, concerns regarding dependency, tolerance, and residual daytime effects have prompted growing interest in non-pharmacological alternatives offering more sustainable benefit [5]. Among non-pharmacological approaches, balneotherapy—involving immersion in mineral or thermal waters—represents a complementary approach that has recently gained interest for improving sleep quality [7]. Balneotherapy should be distinguished from general hydrotherapy (simple heated water immersion) based on the presence of specific mineral compositions that may exert distinct physiological effects beyond thermal stimulation alone [8]. Modern research suggests that balneotherapy may influence sleep through multiple mechanisms, including thermal pathway activation, autonomic nervous system modulation, and reduction in systemic inflammation [7,9,10]. The temperature-regulation hypothesis of sleep initiation posits that body cooling following warm immersion facilitates sleep onset by activating heat loss mechanisms [11,12]. Cutaneous warming promotes vasodilation and subsequent core temperature decline, which aligns with the circadian drop in core temperature that naturally precedes sleep [11]. Studies using heated water baths have demonstrated reduced sleep latency and improved sleep efficiency when administered 1–2 h before bedtime [13]. Sulfurous thermal waters, such as those at Terme di Saturnia (Italy), possess distinctive physicochemical properties beyond temperature alone. These waters contain hydrogen sulfide (H_2_S) concentrations of 14–15 mg/L, calcium sulfate, and other dissolved minerals [14]. Hydrogen sulfide exhibits vasodilatory properties through multiple pathways including activation of ATP-sensitive potassium channels, modulation of nitric oxide pathways, and effects on vascular smooth muscle [15]. Whether transdermal absorption of H_2_S during brief immersion reaches pharmacologically relevant concentrations remains uncertain, and controlled studies comparing sulfurous versus non-sulfurous heated water are lacking. Previous studies on balneotherapy and sleep have reported improvements in subjective sleep quality [7,10], though methodological limitations including heterogeneous interventions and lack of appropriate control groups constrain interpretation [10]. Critically, randomized controlled trials comparing sulfurous mineral water with heated non-mineral water are notably absent from the literature. The conceptual rationale for the present investigation rests on two levels: first, the well-established thermoregulatory model of sleep initiation, which predicts that passive body heating before bedtime may facilitate sleep onset through core temperature decline [11,12], and second, the emerging but unconfirmed hypothesis that sulfurous mineral water may provide additional benefits through H_2_S-mediated vasodilatory and anti-inflammatory pathways [14,15]. This study was designed to generate preliminary real-world data to inform the design of future controlled trials testing these mechanisms, rather than to confirm either hypothesis. The present study aimed to explore changes in self-reported sleep quality following a short-term sulfurous balneotherapy cycle at Terme di Saturnia using the validated Oviedo Sleep Questionnaire in a real-world setting. Given the observational, single-arm design without a control group, findings should be interpreted as hypothesis-generating rather than confirmatory.

## 2. Materials and Methods

### 2.1. Study Design and Setting

This retrospective, single-arm observational study was conducted at Terme di Saturnia (Tuscany, Italy) between June and September 2024. The thermal spring water at Saturnia is characterized by a stable sulfurous calcium–sulfate composition and high mineralization. The main physicochemical parameters of the spring water are summarized in Table 1, based on published hydrogeochemical analyses of the Saturnia source [16]. This characterization is provided to document the mineral profile of the balneotherapy setting and to support reproducibility and comparability with future controlled studies evaluating sulfurous versus non-mineral water immersion. Participants engaged in daily sulfurous thermal water immersion sessions lasting 60–90 min per session, according to routine facility practice and individual tolerance. This study is reported in accordance with the Strengthening the Reporting of Observational Studies in Epidemiology (STROBE) guidelines for observational studies [17]. Due to the retrospective observational nature and use of anonymized data from routine wellness assessments, formal ethics committee approval was waived in accordance with Italian regulations for non-interventional studies. All participants provided informed consent for data use in aggregate form.

### 2.2. Participants

Participants were eligible if they were adults aged 18–75 years who completed a 7–12-day sulfurous balneotherapy cycle and returned both baseline and post-treatment Oviedo Sleep Questionnaire (OSQ) assessments [18]. Individuals were excluded if questionnaires were incomplete or if a known diagnosis of sleep apnea or restless leg syndrome was self-reported. Participants were also excluded when available information indicated acute medical conditions contraindicating immersion or when sleep medication regimens were initiated, discontinued, or modified during the treatment period. Exclusion criteria were based on self-reported information and/or routine facility records available at the time of the wellness assessment. Participants were stratified by baseline insomnia severity into two groups: Group A (OSQ insomnia score ≥ 22) and Group B (OSQ insomnia score < 22). The cut-off value of 22 was selected based on validation studies indicating that scores ≥ 22 correspond to clinically relevant insomnia symptoms [19]. This was a convenience sample comprising all eligible participants who completed balneotherapy cycles during the study period (June–September 2024) and returned complete OSQ assessments. No formal sample size calculation was performed given the exploratory, retrospective nature of the study.

### 2.3. Study Outcomes

Primary outcome: Change in OSQ insomnia sub-scale score from baseline to post-treatment in Group A (participants with baseline sleep disturbances). Secondary outcomes: (1) Change in OSQ sleep satisfaction in Group A. (2) Proportion reporting relaxation effects in Group B. (3). Exploratory descriptive assessment of perceived benefits via open-ended question.

### 2.4. Assessment Instrument

The Oviedo Sleep Questionnaire (OSQ) is a validated instrument for assessing sleep quality and insomnia in clinical and research settings [18]. The OSQ comprises three sub-scales: (1) satisfaction with sleep (scores 1–7, higher scores indicate greater satisfaction); (2) insomnia severity (scores 9–45, higher scores indicate worse insomnia); and (3) hypersomnia (not analyzed in this study).

The OSQ was administered via paper questionnaire at two time-points: (1) baseline assessment on the first day of balneotherapy (retrospective assessment of sleep quality during the previous week), and (2) post-treatment assessment on the final day of the balneotherapy cycle (assessment of sleep quality during the treatment week).

### 2.5. Balneotherapy Protocol

Participants engaged in daily sulfurous thermal water immersion sessions lasting 60–90 min per session, according to routine facility practice and individual tolerance. Immersion occurred in natural thermal pools maintained at 37–38 °C, typically in late afternoon or early evening (15:00–19:00). The broad time window reflects routine clinical practice at the facility and represents a pragmatic element of this real-world study design; however, the unstandardized timing may have introduced variability in any thermoregulatory effects on subsequent sleep, as the interval between immersion and bedtime differed across participants. Participants were free to float, walk slowly, or sit as preferred; no structured exercise protocol was mandated. Participants were advised to maintain adequate hydration and avoid alcohol consumption immediately before or after immersion sessions. No restrictions were placed on other wellness activities available at the facility (e.g., sauna, massage therapy, relaxation areas).

### 2.6. Potential Confounding Factors

Due to the retrospective real-world design, several lifestyle and contextual factors during the spa stay were not systematically recorded prospectively and could not be controlled for in the analysis. These included concurrent wellness activities (e.g., sauna, massage), changes in daily routines, work stress reduction, dietary modifications, and physical activity levels. Limited retrospective data on some contextual factors were collected via participant recall, though these data are subject to recall bias and do not permit quantitative adjustment in the analysis.

### 2.7. Statistical Analysis

Continuous variables were summarized as mean ± SD and categorical variables as frequencies and percentages. Normality of change scores (Δ = post − pre) was assessed using the Shapiro–Wilk test. Given non-normal distributions in several outcomes (Shapiro–Wilk *p* < 0.05) and the ordinal nature of OSQ subscales, non-parametric tests were employed throughout. Pre–post comparisons within each group were performed using the Wilcoxon signed-rank test. Effect sizes were calculated as r = Z/√N, with 95% confidence intervals estimated via bootstrap resampling (1000 iterations), and interpreted according to conventional thresholds: small (r = 0.10–0.29), medium (r = 0.30–0.49), and large (r ≥ 0.50). Between-group comparisons of baseline characteristics were conducted using the Mann–Whitney U test for continuous variables and Fisher’s exact test for categorical variables. Statistical significance was set at *p* < 0.05 (two-tailed). Given the explicitly exploratory, hypothesis-generating nature of this study and the absence of a priori hypotheses, no corrections for multiple comparisons (e.g., Bonferroni) were applied. As a consequence, the reported *p*-values should be interpreted with caution, and the risk of Type I error across multiple endpoints cannot be excluded. Consequently, statistically significant findings should be interpreted with caution and considered hypothesis-generating. All analyses were performed using SPSS version 28.0 (IBM Corp., Armonk, NY, USA). Descriptive data from the open-ended question were categorized using simple content categorization, with discrepancies resolved by consensus. Due to resource constraints, formal thematic analysis and inter-rater reliability assessment were not conducted, limiting the rigor of these exploratory descriptive findings. No sensitivity analyses were performed given the exploratory nature of this initial investigation. The small sample size (n = 76, n = 47 in Group A), retrospective design, and reliance on self-reported confounders of uncertain reliability precluded meaningful multivariate adjustment, which would risk overfitting and potentially misleading precision. Sensitivity analyses and multivariate models controlling for potential confounders (e.g., age, concurrent activities, vacation status) are recommended for future prospective investigations with adequately powered samples.

## 3. Results

### 3.1. Participant Characteristics

**Figure 1:**
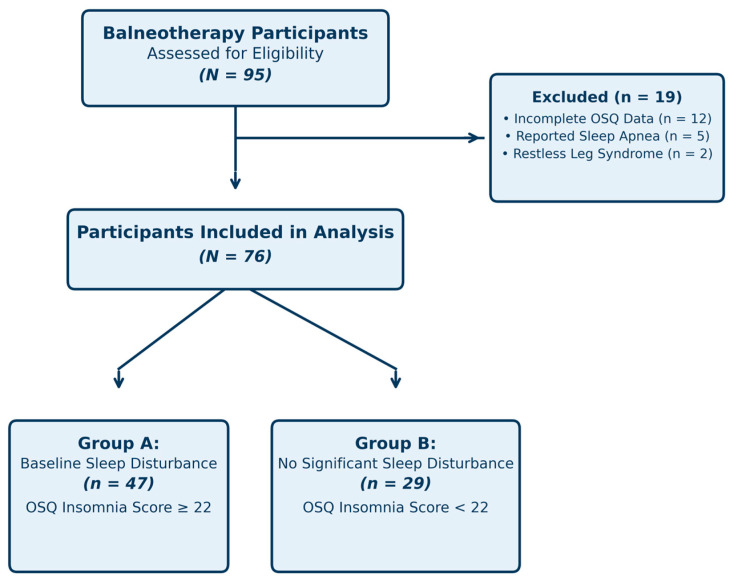
Flow diagram of participant screening, exclusions, and final allocation into Group A (baseline insomnia, OSQ ≥ 22) and Group B (no baseline insomnia, OSQ < 22).

A total of 76 participants met the inclusion criteria and were included in the analysis. The mean age of the sample was 47.3 years (SD = 11.2, range 27–68), and 41 participants (54%) were female. Of the total cohort, 47 participants (62%) were classified as Group A (baseline OSQ insomnia score ≥ 22), whereas 29 participants (38%) were classified as Group B (OSQ insomnia score < 22). Participants completed a mean of 9.4 days of sulfurous balneotherapy (SD = 1.8, range 7–12 days), with daily immersion sessions lasting 60–90 min according to routine facility practice. Participant screening, exclusions, and final group allocation are summarized in Figure 1. Baseline demographic and clinical characteristics are presented in Table 2. Self-reported contextual and lifestyle factors during the spa stay are presented in Table 3. Groups were broadly comparable in demographic characteristics. However, Group A participants tended to be older than Group B (49.1 ± 10.8 vs. 44.6 ± 11.5 years; Mann–Whitney U, *p* = 0.072), a difference that, while non-significant at the conventional α = 0.05 threshold, is clinically relevant given the known association between age and sleep quality and should be considered as a potential confounder. Treatment duration also showed a trend toward between-group difference (*p* = 0.054).

### 3.2. Primary Outcome: Change in Insomnia Severity (Group A)

**Figure 2:**
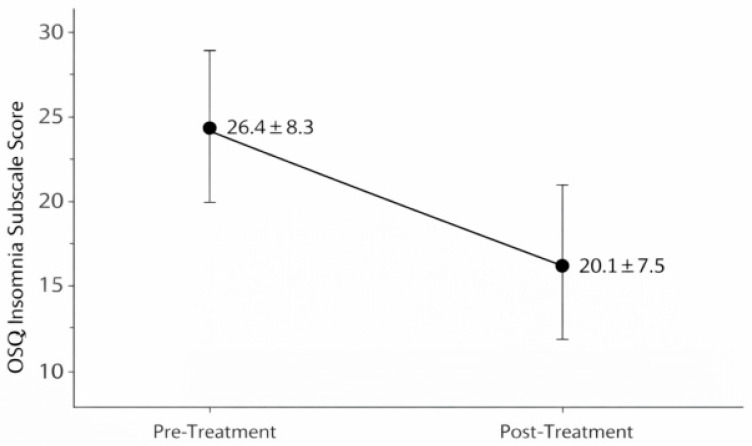
Mean Oviedo Sleep Questionnaire (OSQ) insomnia subscale scores (±SD) at baseline (T0) and post-treatment (T1) in participants with baseline sleep disturbance (Group A, n = 47). Insomnia severity decreased significantly between baseline (T0) and post-treatment (T1) assessments (Wilcoxon signed-rank test, *p* < 0.001).

After a 7–12-day consecutive sulfurous balneotherapy cycle, participants with baseline sleep disturbances (Group A, n = 47, OSQ ≥ 22) showed a significant reduction in insomnia severity scores, decreasing from 26.4 ± 8.3 at baseline to 20.1 ± 7.5 post-treatment (median change: −6.0 points, mean change: −6.3 points, 95% CI: −7.9 to −4.7; Wilcoxon Z = −5.21, *p* < 0.001, effect size r = 0.54, 95% CI: 0.38–0.67). This represents a large effect by conventional standards. For descriptive purposes only, approximately 85% (n = 40) of Group A participants demonstrated score reductions ≥ 3 points. However, this threshold is arbitrary and does not correspond to a validated minimal clinically important difference (MCID), which has not been established for the OSQ insomnia sub-scale in this population, precluding formal clinical significance determination (Figure 2).

### 3.3. Secondary Outcomes

**Figure 3:**
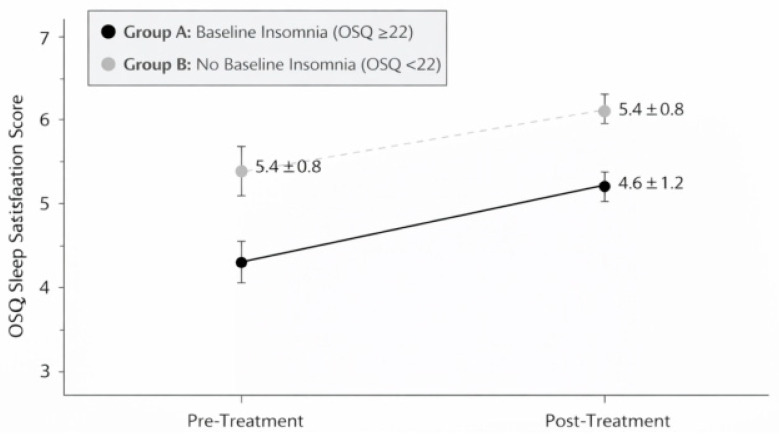
Mean OSQ sleep satisfaction scores (±SD) at baseline (T0) and post-treatment (T1) in Group A (n = 47). Scores improved significantly between baseline (T0) and post-treatment (T1) assessments (*p* < 0.001).

Following the same 7–12-day consecutive sulfurous balneotherapy cycle, sleep satisfaction scores in Group A improved significantly (Figure 3), increasing from 3.2 ± 1.1 at baseline to 4.6 ± 1.2 post-treatment (mean change: +1.4 points, 95% CI: 1.1–1.7; Wilcoxon Z = −5.84, *p* < 0.001, r = 0.60, 95% CI: 0.45–0.72), representing a shift from “somewhat dissatisfied” to “satisfied.” In Group B (no baseline disturbances), neither insomnia scores (14.2 ± 3.8 to 13.5 ± 3.9, Δ = −0.7, 95% CI: −1.8 to 0.4; Z = −1.12, *p* = 0.26, r = 0.21) nor sleep satisfaction scores (5.4 ± 0.8 to 5.7 ± 0.9, Δ = +0.3, 95% CI: −0.1 to 0.7; Z = −1.58, *p* = 0.11, r = 0.29) changed significantly. This likely reflects a ceiling effect, as participants without baseline sleep problems had limited room for measurable improvement.

Notably, 72.4% (21/29; 95% CI: 54.3–85.3) of Group B participants spontaneously reported enhanced relaxation during treatment, despite the absence of statistically significant changes in OSQ scores. All outcomes are summarized in Table 4.

### 3.4. Exploratory Descriptive Participant Feedback

In response to an open-ended question, participants most frequently mentioned improved relaxation (89%), better sleep quality (67%), and reduced muscle tension (58%). A detailed breakdown of responses is provided in Supplementary Table S2. These data represent an unsystematic tally of spontaneous participant responses and do not constitute formal qualitative research. In the absence of systematic coding methodology and inter-rater reliability assessment, they should be interpreted as anecdotal participant feedback only. Responses likely reflect a combination of treatment effects and non-specific factors including vacation enjoyment, reduced stress, and expectancy effects.

## 4. Discussion

This retrospective real-world pre–post observational study explored changes in self-reported sleep quality among spa visitors undergoing a short-term cycle of sulfurous balneotherapy at Terme di Saturnia. Statistically significant pre–post reductions in insomnia severity and improvements in sleep satisfaction were observed in participants with baseline sleep disturbance.

However, these changes occurred in the context of a multi-component spa vacation, with the majority of participants on vacation and reporting reduced work-related stress and concurrent wellness activities (Table 3). The single-arm design without control group does not allow causal attribution of the observed improvements to any specific component of the experience, including sulfurous water immersion. Non-specific factors—the vacation effect, stress reduction, environmental change, and regression to the mean—represent plausible alternative explanations. The observed changes must also be interpreted in light of substantial non-specific influences. Spa-based interventions occur within a context strongly associated with stress reduction, schedule regularization, increased physical activity, and exposure to restorative natural environments. Such contextual effects are well documented: even short-term holidays have been shown to improve perceived well-being and reduce stress-related symptoms, which may secondarily enhance sleep quality [20]. Moreover, self-reported sleep measures have been shown to capture broader subjective and psychological dimensions beyond objective sleep parameters. In a longitudinal study of 249 individuals with depression monitored over 13 weeks, Akre et al. demonstrated that self-reported and physiologically measured sleep quality assess distinct constructs, with weak correlations between the two modalities and self-reported measures showing stronger associations with psychological symptoms [21]. The exclusive reliance on self-report in the present study may therefore have amplified the apparent magnitude of improvement, as participants’ responses likely integrated the overall positive experience of the spa environment rather than reflecting changes in sleep architecture alone. In addition, placebo and expectancy effects are particularly pronounced in insomnia research, with response rates in placebo arms frequently reaching 30–50% [22]. Regression to the mean may further contribute, especially in participants selected on the basis of elevated baseline symptom scores.

Sleep initiation and maintenance are closely linked to thermoregulatory processes, particularly the decline in core body temperature that normally precedes sleep onset. Experimental evidence indicates that passive body heating may facilitate subsequent heat dissipation through peripheral vasodilation, thereby promoting sleep propensity. In a systematic review and meta-analysis, warm water bathing performed approximately 1–2 h before bedtime was associated with shorter sleep onset latency and improved self-reported sleep quality, suggesting that thermal stimulation alone may exert measurable effects on sleep regulation [19].

These findings are further supported by physiological data in older adults showing that hot-water bathing before bedtime increases the distal–proximal skin temperature gradient, a marker of enhanced heat loss correlating with improved sleep initiation [23,24]. Thus, even in the absence of mineral-specific effects, immersion in warm thermal pools may plausibly contribute to improved sleep through thermophysiological pathways. Beyond thermal mechanisms, sulfurous waters contain hydrogen sulfide (H_2_S), a gaseous signaling molecule with recognized vasodilatory and anti-inflammatory properties [15]. Preclinical and clinical evidence suggests that H_2_S may exert additional effects relevant to sleep regulation, including hypothermia induction via TRPA1 channel activation [25], improvement of hemorheological parameters beyond thermal stimulation alone [26], and modulation of cellular signaling pathways through transdermal absorption during balneotherapy [14]. However, it remains uncertain whether transdermal exposure during balneotherapy produces systemic concentrations sufficient to induce clinically meaningful effects. These putative H_2_S-mediated effects remain entirely hypothetical in the context of the present study, which was not designed to test mineral-specific contributions. The present study provides no evidence that sulfurous mineral water composition confers specific benefits beyond those attributable to warm water immersion, the spa environment, or the vacation context. Any mineral-specific therapeutic contribution therefore remains speculative and awaits evaluation through controlled trials comparing sulfurous with non-mineral heated water.

Following these mechanistic considerations, the present findings are broadly consistent with the limited but growing literature suggesting that spa-based thermal interventions may be associated with improvements in subjective sleep outcomes. A recent systematic review examining hydrotherapy, spa therapy, and balneotherapy concluded that these interventions may improve sleep quality, although the certainty of evidence remains low due to heterogeneity and frequent methodological limitations, including small sample sizes and a lack of adequate control groups [10]. Similarly, another systematic review highlighted potential benefits of balneotherapy on sleep parameters but emphasized that most available studies are observational and do not allow attribution of effects to mineral composition versus non-specific contextual factors [8].

In line with this, Castelli et al. reported that spa stays combining daytime physical activity and balneotherapy were associated with improved sleep and recovery, suggesting that multimodal lifestyle changes during spa exposure may contribute substantially to perceived benefits [7]. Therefore, the improvements observed in the present cohort may reflect not only immersion effects but also broader behavioral and environmental modifications occurring during the treatment period. This interpretation is supported by recent evidence from a survey study of 315 healthy adults demonstrating that workday sleep duration is a significant determinant of self-reported sleep quality, which in turn correlates negatively with anxiety, depression symptoms, and perceived workload; notably, increased physical activity and absence of smoking were independently associated with better sleep quality [27]. In the present cohort, 79% of participants reported reduced work-related stress and 63% increased physical activity during the spa stay (Table 3), suggesting that these lifestyle modifications alone may have contributed substantially to the observed sleep improvements. Importantly, the present design cannot determine whether sulfurous mineral composition provides benefits beyond those expected from passive heating and the spa environment itself. Warm water immersion alone has demonstrated measurable effects on sleep onset and subjective sleep quality, and the absence of a heated non-mineral water comparator precludes isolation of any incremental sulfur-specific contribution [19]. Thus, any interpretation of hydrogen sulfide-mediated mechanisms must remain hypothetical.

## 5. Limitations

Several important limitations should be considered when interpreting these findings. First, the retrospective single-arm pre–post design does not allow causal inference. Without a control or comparative group (e.g., heated non-mineral water immersion, wait-list controls, or usual care), the observed improvements cannot be attributed specifically to sulfurous balneotherapy. Non-specific influences such as placebo effects, expectancy, regression to the mean, and the well-described “vacation effect” may have contributed substantially to the changes in self-reported sleep outcomes.

Second, several relevant confounding factors were not systematically measured. Participants were immersed in a complex wellness context that may have included reduced work-related stress, increased outdoor exposure, lifestyle modifications, physical activity, and concurrent spa services (e.g., sauna or massage). Because these factors were not controlled or quantified prospectively, the specific contribution of mineral water properties cannot be isolated. Third, outcomes relied exclusively on subjective self-report measures. Although the Oviedo Sleep Questionnaire is validated, the absence of objective sleep assessments (such as actigraphy or polysomnography) limits the ability to confirm whether perceived improvements corresponded to measurable changes in sleep architecture or efficiency. Fourth, selection bias is likely. Participants were self-selected spa visitors, potentially representing a health-conscious population with positive expectations toward balneotherapy. Furthermore, the sample was demographically homogeneous (predominantly middle-aged, with sufficient socioeconomic resources to access a private thermal facility), and data on comorbidities, ethnicity, education level, and socioeconomic status were not systematically collected, further limiting generalizability. This limits the generalizability of the findings to broader clinical insomnia populations. Fifth, post-treatment assessments were performed immediately at the end of the balneotherapy cycle, and no longitudinal follow-up was available. Therefore, the durability of the reported sleep improvements beyond the treatment or vacation period remains unknown.

Sixth, qualitative findings were derived from open-ended responses without formal thematic analysis or inter-rater reliability procedures. These results should therefore be interpreted as supportive contextual information rather than robust qualitative evidence. Furthermore, although between-group differences in age and treatment duration did not reach statistical significance, the observed trends (*p* = 0.072 and *p* = 0.054, respectively) suggest that age may represent a potential confounder, as sleep architecture and insomnia prevalence are known to change with ageing. The absence of multivariate adjustment for age and other potential confounders represents a further limitation of this exploratory analysis. The limited sample size (n = 47 in Group A) precluded reliable multivariate modelling without risk of overfitting. Finally, the broad immersion time window (15:00–19:00) was not standardized, introducing potential variability in the thermoregulatory stimulus relative to bedtime, which may have influenced sleep outcomes heterogeneously across participants.

## 6. Conclusions

This exploratory real-world observational study documents statistically significant pre–post improvements in self-reported insomnia severity and sleep satisfaction among spa visitors with baseline sleep disturbance who underwent a short-term sulfurous balneotherapy cycle (7–12 consecutive days). Participants without baseline sleep disturbance showed stable quantitative OSQ scores, consistent with ceiling effects, but frequently reported enhanced relaxation and sleep-related comfort during the spa stay. However, the absence of a control group and unmeasured confounders preclude causal inference. Future randomized controlled trials comparing sulfurous mineral water with heated non-mineral water immersion are needed to isolate any specific therapeutic contribution of sulfurous thermal waters.

## Figures and Tables

**Table 1:** Physicochemical profile of Saturnia sulfurous thermal water at source, reported to characterize the mineral composition of the balneotherapy intervention setting.

Parameter	Value (Approx.)
Temperature	37.5 °C
Conductivity	~3110 µS/cm
Hydrochemical type	Calcium–sulfate water
Sulfate (SO_4_^2−^)	~1540 mg/L
Calcium (Ca^2+^)	~593 mg/L
Boron (B)	~18 mg/L

**Table 2:** Baseline demographic and clinical characteristics of the study participants.

Characteristic	Total (n = 76)	Group A (OSQ ≥ 22) (n = 47)	Group B (OSQ < 22) (n = 29)	*p*-Value
Age, years (mean ± SD)	47.3 ± 11.2	49.1 ± 10.8	44.6 ± 11.5	0.072
Female sex, n (%)	41 (54%)	27 (57%)	14 (48%)	0.442
Treatment duration, days (mean ± SD)	9.4 ± 1.8	9.6 ± 1.7	9.1 ± 1.9	0.054
First-time visitor, n (%)	28 (37%)	15 (32%)	13 (45%)	0.256
Baseline insomnia score (mean ± SD)	21.4 ± 7.9	26.4 ± 8.3	14.2 ± 3.8	<0.001
Baseline sleep satisfaction (mean ± SD)	3.9 ± 1.4	3.2 ± 1.1	5.4 ± 0.8	<0.001

Group A: baseline OSQ insomnia score ≥ 22. Group B: baseline OSQ insomnia score < 22. *p*-values from Mann–Whitney U test (continuous variables) or Fisher’s exact test (categorical variables). No participants in the final analyzed sample (n = 76) had missing data for any variable presented in this table.

**Table 3:** Self-reported contextual and lifestyle factors potentially influencing sleep outcomes during the spa stay.

Contextual Factor	Total (n = 76)	Group A (n = 47)	Group B (n = 29)
Participants on vacation during treatment, n (%)	68 (89%)	42 (89%)	26 (90%)
Increased outdoor time reported, n (%)	71 (93%)	44 (94%)	27 (93%)
Concurrent wellness activities (sauna/massage), n (%)	42 (55%)	27 (57%)	15 (52%)
Reported reduced work-related stress, n (%)	60 (79%)	38 (81%)	22 (76%)
Increased physical activity (walking/swimming), n (%)	48 (63%)	30 (64%)	18 (62%)
Dietary modifications (reduced caffeine/alcohol), n (%)	39 (51%)	25 (53%)	14 (48%)

Data derived from retrospective participant responses and should be interpreted cautiously due to recall bias and incomplete measurement of confounders.

**Table 4:** Pre–post changes in OSQ insomnia and sleep satisfaction scores following sulfurous balneotherapy.

Outcome	Baseline (T0) Mean ± SD	Post-Treatment (T1) Mean ± SD	Mean Change (Δ)	95% CI	*p*-Value	Effect Size r (95% CI)
Group A (OSQ ≥ 22), n = 47						
Insomnia score (9–45)	26.4 ± 8.3	20.1 ± 7.5	−6.3	−7.9 to −4.7	<0.001	0.54 (0.38–0.67)
Sleep satisfaction (1–7)	3.2 ± 1.1	4.6 ± 1.2	+1.4	1.1 to 1.7	<0.001	0.60 (0.45–0.72)
Group B (OSQ < 22), n = 29						
Insomnia score (9–45)	14.2 ± 3.8	13.5 ± 3.9	−0.7	−1.8 to 0.4	0.26	0.21 (0.01–0.40)
Sleep satisfaction (1–7)	5.4 ± 0.8	5.7 ± 0.9	+0.3	−0.1 to 0.7	0.11	0.29 (0.08–0.48)

*p*-values from Wilcoxon signed-rank tests. Effect size r calculated as r = Z/√N.

## Data Availability

The data presented in this study are available on request from the corresponding author. The data are not publicly available due to privacy and confidentiality restrictions concerning individual health information collected during wellness assessments at a private healthcare facility.

## Supplementary Materials

The following supporting information can be downloaded at: https://www.mdpi.com/article/10.3390/healthcare14060782/s1, Table S1: Complete STROBE Checklist. Table S2. Self-reported benefits from sulfurous thermal water treatment: exploratory descriptive participant feedback (N = 76).
