# Supplementary material for: Short-Term Sulfurous Balneotherapy and Self-Reported Sleep Quality: An Exploratory Retrospective Real-World Pre–Post Observational Study at Terme di Saturnia (Italy)
Source: Healthcare (Basel). 2026 Mar 19;14(6):782. doi: 10.3390/healthcare14060782 (PMC13026361; doi:10.3390/healthcare14060782)
Supplement: Supplementary file 1 [file healthcare-14-00782-s001.zip › healthcare-4146492-supplementary-table S2.pdf]

healthcare

Table S2. Self-reported benefits from sulfurous thermal water treatment: exploratory descriptive participant feedback (N = 76).

Reported Benefit	n	%	95% CI (%)
Improved relaxation	68	89.5	80.6–94.6
Better sleep quality	51	67.1	55.9–76.6
Reduced muscle tension	44	57.9	46.7–68.4
Enhanced mood	39	51.3	40.3–62.2
Improved skin condition	27	35.5	25.7–46.7

Data derived from an open-ended question, "What benefits did you experience from the thermal water treatment?", administered at the end of the balneotherapy cycle. Multiple responses per participant were permitted.

95% confidence intervals calculated using the Wilson score method for binomial proportions.

Important caveats: These data represent an unsystematic tally of spontaneous participant responses and do not constitute formal qualitative research. Responses were categorized by simple content classification without formal thematic analysis or inter-rater reliability assessment. Given the retrospective self-report format and absence of systematic coding methodology, these findings should be interpreted as anecdotal participant feedback only. Reported benefits likely reflect a combination of specific treatment effects and non-specific factors, including vacation enjoyment, stress reduction, environmental change, and expectancy effects.

Abbreviations: CI, confidence interval; n, number of participants reporting each benefit.
